# Disconnect between EMT and metastasis in pancreas cancer

**DOI:** 10.18632/oncotarget.5720

**Published:** 2015-09-18

**Authors:** Martin C. Whittle, Sunil R. Hingorani

**Affiliations:** Clinical Research Division and Public Health Sciences Division, Fred Hutchinson Cancer Research Center and Division of Medical Oncology, University of Washington School of Medicine, Seattle, WA

**Keywords:** Chromosome Section, RUNX3, SMAD4, circulating tumor cells, TGFβ

Early and widespread metastasis remains a major challenge to the effective treatment of pancreatic ductal adenocarcinoma (PDA). With the current dearth of screening options contributing to late diagnoses, the majority of patients present with and ultimately succumb to metastatic disease [1]. The program of epithelial to mesenchymal transition (EMT) is often implicated in the metastatic process of cancers, including PDA. The canonical “master regulator” of EMT is the TGFβ pathway: TGFβ induces dimerization of surface receptors promoting phosphorylation of SMAD2/3, followed by association of phospho-SMAD2/3 with SMAD4 and nuclear transport of the trimeric complex, resulting in upregulation of mesenchymal markers, such as SNAIL1 and vimentin, and downregulation of epithelial markers such as E-cadherin [2]. Despite the perceived requisite ability to undergo EMT for cancer metastasis, inactivation of *SMAD4* is a frequent event in PDA progression and strongly associated with enhanced metastasis while also being a critical component of the TGFβ signaling pathway that potently induces EMT [1]. We have recently shown that pancreas-specific homozygous deletion of *SMAD4* in the *KPC* mouse model of PDA abrogates TGFβ-induced EMT of cancer epithelia, but does not impair metastasis [3]. We found that the biphasic regulation of the RUNX3 transcription factor - up in *SMAD4^+/+^* and *SMAD4^−/−^* and down in *SMAD4^+/−^* PDA - mirrors the metastatic potential of PDA cells with these distinct genotypes, which is also independent of the capacity to undergo EMT. RUNX3 induces the expression of secreted proteins such as SPP1 and COL6A1 that stimulate migration and condition a metastatic niche, both of which may act independently of the EMT-competency of cancer cells. These findings suggest that EMT is dispensable for PDA metastasis and support the possibility of distinct EMT-competent and EMT-incompetent mechanisms of PDA dissemination.

The migration and intravasation of cancer cell clusters are now well established as an alternative model to the classically envisioned single circulating tumor cell (CTC) for the origin of metastatic deposits [4, 5]. In fact, although CTC clusters or tumor microemboli appear to be much rarer than isolated CTC, their efficiency at seeding metastases is estimated to be 20-50 times higher due, in part, to the evasion of anoikis and increased likelihood of becoming lodged in narrow blood vessels [4]. The role that EMT plays in the dissemination of either single or clustered CTC is unclear, however, and is hampered by technical challenges impeding the continuous observation of the complete metastatic process. That increased mesenchymal markers have been observed in clustered compared to single CTC does not imply that intravasation of CTC clusters requires EMT; initation of EMT in CTC clusters may also occur after bloodstream entry by cluster-associated platelets that secrete high levels of TGFβ [5]. CTC aggregates comprise a higher percentage (6%) of overall CTC detected in pancreas compared to breast, prostate and lung cancers [6], and the association of enhanced metastatic burden with either completely intact or completely inactivated *SMAD4* vs. decreased metastasis in the heterozygous state seems to suggest that partially attenuated TGFβ signaling suppresses metastatic potential, perhaps by shifting the CTC burden from one phenotype to another. These are readily testable hypotheses.

**Figure 1:**
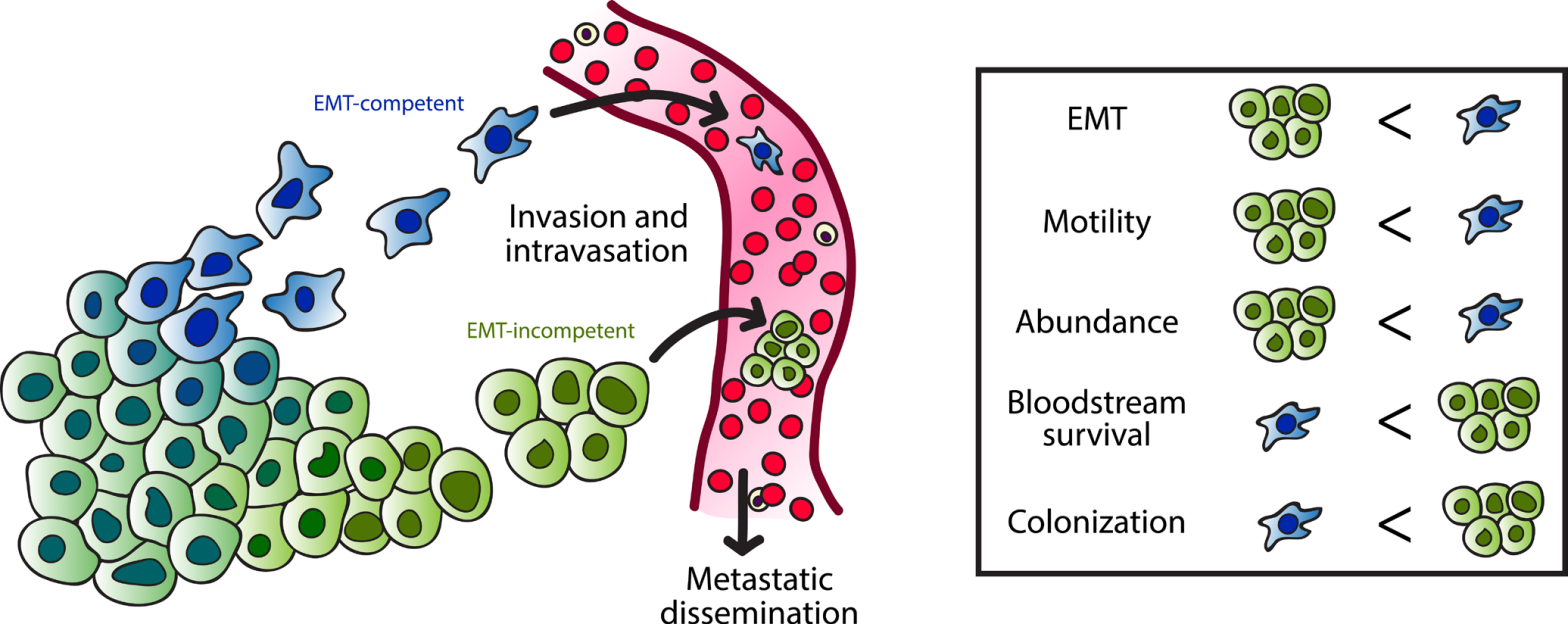
EMT-competent and incompetent mechanisms of metastasis Invasion and intravasation of pancreas cancer cells can occur by EMT-dependent or EMT-independent mechanisms. EMT-competent cells likely disseminate as individual migrating cells, whereas EMT-incompetent cells may favor clustered migration. Individual circulating tumor cells (CTC) are more abundant and exhibit increased motility compared to CTC clusters, however clustered CTC are more likely to survive in the bloodstream and colonize distant sites.

It is evident that tumor cells in circulation must exhibit a high degree of plasticity to withstand mechanical and chemical forces that threaten their survival. Though a concerted program of EMT may be dispensable for single or clustered cancer cell migration and metastatic dissemination, successful colonization surely requires the acquisition of features that enhance motility and survival. An undue emphasis on the *in vitro* phenomenon of EMT and its bearing on the metastatic process fuels a debate that may distract as much as it illuminates. An ability to undergo EMT is neither necessary nor sufficient for metastasis. Elucidation of the diverse mechanisms underlying successful metastatic colonization will be vital for improving cancer therapies, and this endeavor may benefit from shedding a dependence on the term “EMT” as a shortcut for metastatic potential and replacing or refining it with more precise language to describe the intricate biology of metastasis.
